# Status Report on the First Round of the Development of the Advanced Encryption Standard

**DOI:** 10.6028/jres.104.027

**Published:** 1999-10-01

**Authors:** James Nechvatal, Elaine Barker, Donna Dodson, Morris Dworkin, James Foti, Edward Roback

**Affiliations:** National Institute of Standards and Technology, Gaithersburg, MD 20899-0001

**Keywords:** Advanced Encryption Standard (AES), cryptography, cryptanalysis, cryptographic algorithms, encryption

## Abstract

In 1997, the National Institute of Standards and Technology (NIST) initiated a process to select a symmetric-key encryption algorithm to be used to protect sensitive (unclassified) Federal information in furtherance of NIST’s statutory responsibilities. In 1998, NIST announced the acceptance of 15 candidate algorithms and requested the assistance of the cryptographic research community in analyzing the candidates. This analysis included an initial examination of the security and efficiency characteristics for each algorithm. NIST has reviewed the results of this research and selected five algorithms (MARS, RC6™, Rijndael, Serpent and Twofish) as finalists. The research results and rationale for the selection of the finalists are documented in this report. The five finalists will be the subject of further study before the selection of one or more of these algorithms for inclusion in the Advanced Encryption Standard.

## 1. Overview of the Development Process for the Advanced Encryption Standard and Summary of Round 1 Evaluations

The National Institute of Standards and Technology (NIST) has been working with industry and the cryptographic community to develop an Advanced Encryption Standard (AES). The overall goal is to develop a Federal Information Processing Standard (FIPS) that specifies an encryption algorithm(s) capable of protecting sensitive (unclassified) government information well into the next century. The algorithm(s) is expected to be used by the U.S. Government and, on a voluntary basis, by the private sector.

On January 2, 1997, NIST announced the initiation of an effort to develop the AES [16] and made a formal call for algorithms on September 12, 1997 [17]. The call stipulated that the AES would specify an unclassified, publicly disclosed encryption algorithm(s), available royalty-free, worldwide. In addition, the algorithm(s) must implement symmetric key cryptography as a block cipher and (at a minimum) support a block size of 128 bits and key sizes of 128 bits, 192 bits, and 256 bits.

On August 20, 1998, NIST announced its acceptance of 15 AES candidate algorithms at the First AES Candidate Conference (AES1). These algorithms had been submitted by members of the cryptographic community from around the world. At that conference and in a published notice [18], NIST solicited public comments on the candidates. A Second AES Candidate Conference (AES2) was held in March 1999 to discuss the results of the analysis conducted by the global cryptographic community on the candidate algorithms. The public comment period on the initial review of the algorithms closed on April 15, 1999.

Using the analyses and comments received, NIST selected 5 finalist algorithms from the 15. The selected algorithms are MARS, RC6™, Rijndael, Serpent and Twofish. These algorithms will receive further analysis during a second, more in-depth review period prior to the selection of the final AES algorithm(s).

The remainder of Sec. 1 summarizes the evaluation process and briefly describes the selected algorithms. Section 2 of this report contains the technical details of the analyses conducted during Round 1.

### 1.1 Evaluation Criteria

In the call for candidate algorithms [17], NIST specified the evaluation criteria that would be used to compare the candidate algorithms. These criteria were developed from public comments to [16] and from the discussions at a public AES workshop held on April 15, 1997 at NIST.

The evaluation criteria are divided into three major categories: 1) Security, 2) Cost, and 3) Algorithm and Implementation Characteristics. Security is the most important factor in the evaluation, and it encompasses features such as: resistance of the algorithm to cryptanalysis, soundness of its mathematical basis, randomness of the algorithm output, and relative security as compared to other candidates.

Cost is a second important area of evaluation that encompasses licensing requirements, computational efficiency (speed) on various platforms, and memory requirements. Since one of NIST’s goals is that the final AES algorithm(s) be available worldwide on a royalty-free basis, intellectual property claims and potential conflicts must be considered in the selection process. The speed of the algorithms on a wide variety of platforms must also be considered. During Round 1, the focus was primarily on the speed associated with 128 bit keys. Additionally, memory requirements and constraints for software implementations of the candidates are important considerations.

The third area of evaluation is algorithm and implementation characteristics such as flexibility, hardware and software suitability, and algorithm simplicity. Flexibility includes the ability of an algorithm: 1) to handle key and block sizes beyond the minimum that must be supported, 2) to be implemented securely and efficiently in many different types of environments, and 3) to be implemented as a stream cipher, hashing algorithm, and to provide additional cryptographic services. It must be feasible to implement an algorithm in both hardware and software, and efficient firmware implementations are advantageous. The relative simplicity of an algorithm’s design is also an evaluation factor.

### 1.2 Results From Round 1

The Round 1 public review extended from the official announcement of the 15 AES candidates on August 20, 1998, at AES1 [36] until the official close of the comment period on April 15, 1999. During Round 1, many members of the global cryptographic community supported the AES development effort by analyzing and testing the 15 AES candidates.

NIST facilitated and focused the discussion of the candidate algorithms by providing an electronic discussion forum that was used to comment on the candidates, discuss relevant AES issues, inform the public of new analysis results, etc. This discussion forum is located at [1]. The AES home page [2] has served as a tool to disseminate information such as papers for AES2 and other Round 1 public comments.

Twenty-eight papers were submitted to NIST for consideration for AES2. Twenty-one of those papers were presented at AES2 as part of the formal program, and several of the remaining seven were also presented during an informal session at that conference. All of the submitted papers were posted on the AES home page [2] several weeks prior to AES2 in order to promote informed discussions at the conference.

AES2 gave members of the global cryptographic community a chance to present and discuss the analysis that had been performed on the AES candidates during Round 1, as well as to discuss other important topics relevant to the AES development effort. A summary of AES2 presentations and discussions can be found in Ref. [15]. In addition to the AES2 papers, NIST received 56 sets of public comments during Round 1 regarding the candidate algorithms. All of these comments were made publicly available on the AES home page [2] on April 19, 1999.

NIST performed an analysis of mathematically optimized ANSI C and Java™ implementations^1^ of the candidate algorithms that were provided by the submitters prior to the beginning of Round 1. Additionally, extensive statistical testing was performed by NIST on all of the candidates. The testing of ANSI C implementations focused on the speed of all fifteen candidates on various desktop systems, using different combinations of processors, operating systems, and compilers. The submitters’ Java code was tested for speed and memory usage on a desktop system, and other features of the code were measured as well. Statistical testing was performed on all fifteen candidates to determine if the algorithms generate output that is statistically indistinguishable from truly random data. The results of this testing is available on the AES home page [2].

### 1.3 Selection Process Prior to Round 2

At the conclusion of the Round 1 public review, NIST established an internal AES technical review team to recommend algorithms for Round 2 evaluations. The members of this team included NIST employees who had been engaged in: 1) reviewing the algorithms, 2) reviewing the public comments on the candidates, 3) selecting papers for AES2, 4) conducting NIST’s efficiency and randomness testing, 5) attending and presenting information at the AES conferences, and 6) managing the AES development process. The team met several times over the course of 2 months to develop their consensus position.

During the evaluation process, all comments, papers, verbal comments at conferences, NIST studies, reports and proposed modifications were considered. Each candidate was discussed relative to the announced evaluation criteria and other pertinent criteria suggested during the public analysis. The review of each algorithm included a methodical evaluation of: 1) security (including any known attacks or weaknesses), 2) efficiency (both speed and memory usage), 3) flexibility (implementation on low- and high-end smart cards; support of additional key and block sizes—including whether the reference code actually supported the additional key sizes; suitability for use as a pseudo-random number generator, hashing algorithm, etc.; and whether or not encryption and decryption were the same procedure), 4) algorithm simplicity, and 5) other issues that were discussed in the received public comments. Refer to Sec. 2 for the detailed evaluation of all 15 algorithms.

Although it was considered, the team readily agreed that it was not possible to conduct a thorough quantitative selection of the finalists. For example, comments were not received regarding the security analysis of some candidates, whereas other algorithms were reported as “broken.” Since security is considered the most important evaluation criteria, the AES review team made a first cut of the candidates based on security then proceeded with the other selection criteria. As a result of this evaluation process, the five candidates with superior characteristics were selected as finalists for Round 2 evaluation.

It is important to note that the selection of an algorithm as a finalist does not constitute endorsement by NIST of the algorithm or its security. Similarly, the nonselection of an algorithm is not necessarily to be taken as a statement about the algorithm’s quality, security, efficiency or other characteristics.

### 1.4 Round 2 Finalists

The five candidates that NIST selected for further analysis in Round 2 are MARS, RC6, Rijndael, Serpent, and Twofish.

No significant security vulnerabilities were found for these candidates during the Round 1 analysis, and each of these algorithms constitutes potentially superior technology. Below is a summary of each of the finalist candidates in alphabetical order; profiles and overall assessments for all fifteen Round 1 candidates can be found in Secs. 2.6 and 2.7.

MARS incorporates its “cryptographic core” into an innovative, heterogeneous overall structure. It also features a wide variety of operations, including the technique of rotating digits by a varying number of places that is determined by both the data and the secret key. Consequently, while MARS performs well in general, it performs particularly well on computer platforms that support its rotation and multiplication operations efficiently. NIST accepted a modification to MARS for Round 2 (proposed by the submitter) that should improve its ability and flexibility to function in some memory-constrained environments, such as low-end smart cards. MARS was submitted to the AES development effort by the International Business Machines Corporation.

RC6 is an algorithm that is simple enough to memorize, and should be easy to implement compactly in both software and hardware. Its simplicity also should facilitate its further security analysis in Round 2, which is assisted by the analysis of its predecessor, RC5. RC6 does not use substitution tables; instead, the principal engine for its security is the technique of rotating digits by a varying number of places that is determined by the data. In general, RC6 is fast, and it is particularly fast on platforms that support its rotation and multiplication operations efficiently; its key setup is also fast. RC6 was submitted to the AES development effort by RSA Laboratories.

Rijndael performs excellently across all considered platforms. Its key setup is fast, and its memory requirements are low, so it also should perform well in hardware and in memory-constrained environments. The straightforward design and the conservative choice of operations should facilitate its further analysis, and the operations should be relatively easy to defend against certain attacks on physical implementations. Even though parallel processing was not considered during the Round 1 selection process by the AES review team, Rijndael has the potential of benefiting from advances in computer processors that allow many instructions to be executed in parallel. Rijndael was submitted to the AES development effort by Joan Daemen and Vincent Rijmen.

Serpent is ultra-conservative in its security margin: the designers chose to use twice as many iterations as they believed secure against currently known attacks. Consequently, Serpent’s performance is relatively slow compared to the other four finalists; however, in some settings this should be mitigated by the efficiency of optimized implementations using what the submitters call the “bitslice” mode, for which the algorithm was specially designed. Serpent should fit well in hardware (with potential tradeoffs of speed versus space) and in memory-constrained environments. The straightforward design and the conservative choice of operations should facilitate further analysis of this candidate, and the operations should be easy to defend against certain attacks on physical implementations. Serpent was submitted to the AES development effort by Ross Anderson, Eli Biham, and Lars Knudsen.

Twofish exhibits fast and versatile performance across most platforms; it also should perform well both in hardware and in memory-constrained environments. It features variable substitution “tables” that depend on the secret key. The submitters believe that such tables generally offer greater security than tables with fixed values. The possibility of pre-computing these tables to varying degrees helps Twofish to offer a wide variety of performance tradeoffs: depending on the setting, Two-fish can be optimized for speed, key setup, memory, code size in software, or space in hardware. Twofish was submitted to the AES development effort by Bruce Schneier, John Kelsey, Doug Whiting, David Wagner, Chris Hall, and Niels Ferguson.

### 1.5 Next Steps

With the announcement of the finalists, NIST formally opens the “Round 2” public evaluation process and solicits comments on the remaining algorithms through May 15, 2000. Comments and analysis are actively sought by NIST on any aspect of the candidate algorithms, including—but not limited to—the following topics: cryptanalysis, intellectual property, crosscutting analyses of all of the AES finalists, selection and use of multiple AES algorithms, overall recommendations and implementation issues.

NIST is providing an opportunity for the sponsors of the AES finalists to revise the ANSI C and Java implementations of their algorithms. NIST intends to make these implementations available (via CDROM) within two months of the beginning of Round 2.

Near the end of Round 2, NIST will sponsor the Third AES Candidate Conference (AES3)—an open, public forum for discussion of the analyses of the AES finalists. Submitters of the AES finalists will be invited to attend and engage in discussions regarding comments on their algorithms. AES3 will be held April 13–14, 2000 in New York, NY. Registration and logistical information will be posted on the AES home page [2]. Proposed papers for this conference are due to NIST by January 15, 2000.

Following the close of the Round 2 public analysis period on May 15, 2000, NIST intends to study all available information and propose the AES, which will incorporate one or more AES algorithms selected from the finalists. The AES will be announced as a proposed Federal Information Processing Standard (FIPS) which will be published for public review and comment. Following the comment period, the standard will be revised, as appropriate, by NIST in response to those comments. A review, approval, and promulgation process will then follow. If all steps of the AES development process proceed as planned, it is anticipated that the standard will be completed by the summer of 2001.

## 2. Technical Details of the Round 1 Analysis

### 2.1 Abbreviations

The following abbreviations will be used in the remainder of the report: DFC = Decorrelated Fast Cipher, HPC = Hasty Pudding Cipher.

### 2.2 Organization of Section 2

The analysis in this paper is predicated upon the original specifications and code submitted for the candidates prior to the beginning of Round 1, in response to NIST’s call for candidate algorithms [17]. General security issues pertaining to these candidates are discussed in Sec. 2.3. Another area which received a lot of attention during Round 1 were general efficiency issues, which follow in Sec. 2.4. Section 2.5 discusses issues related to smart cards. Profiles of the candidates are presented in Sec. 2.6, summarizing information from Round 1 for each individual candidate. Section 2.7 gives overall assessments for the candidates, and the decision regarding each is presented. Modification proposals for four of the candidates are addressed in Sec. 2.8.

### 2.3 General Security

Security was the foremost concern in evaluating the candidates. Some security issues regarding implementation pertain to smart cards and similar environments; these are discussed in Sec. 2.5. General issues are discussed below.

#### 2.3.1 Major Attacks

The following are the major attacks against the candidates found to date:

- DEAL: in Ref. [30], it is claimed that DEAL-192 is no more secure than DEAL-128.
- FROG: in Ref. [41], it is claimed that a fraction (about 2^−30^) of the key space is weak; for these keys, attacks with a work factor of about 2^56^ are feasible. It is also claimed that diffusion is weaker in the reverse direction.
- HPC: in Ref. [14], it is claimed that for 128 bit keys, 1 in 256 keys has 2^30^ equivalent keys.
- LOKI97: in Ref. [25], it is claimed that attacks exist using only about 2^56^ chosen or known plaintexts.
- MAGENTA: in Ref. [7], it is claimed that attacks using only about 2^64^ chosen plaintexts are feasible; [7] also claims an attack using 2^33^ known plaintexts and 2^97^ steps of analysis.

NIST has verified the attacks on MAGENTA and LOKI97, as well as equivalent keys of HPC, whose existence has been confirmed via statistical testing [32]. Coincidentally, the five attacked candidates were also among the slowest candidates. Combined with weak or questionable security, the conclusion was that none of these was a strong candidate for the AES, as discussed for each candidate in Secs. 2.6 and 2.7.

#### 2.3.2 Lesser Attacks

Some attacks of a lesser magnitude have also been found:

- CRYPTON: in Ref. [8], it is claimed that there are 2^32^ weak 256 bit keys. The same claim is made independently in Ref. [40]. In addition, [13] claims an attack on a 6-round version of CRYPTON.
- DEAL: in Ref. [24], equivalent keys are claimed for a fraction (2^−64^) of the 192 bit and 256 bit key spaces.
- DFC: in Ref. [26], an attack on a 6-round version is claimed, using about 2^70^ chosen ciphertexts and about 2^129^ evaluations of the round function.
- E2: in Ref. [31], attacks on 9- and 10-round versions are claimed.
- SAFER+: in Ref. [23], a related-key attack and a meet-in-the-middle attack are claimed against SAFER+ for the 256 bit key size.

None of the above attacks was deemed by NIST to be serious enough to be a sole factor in deciding admission to Round 2. All of the candidates mentioned above were eliminated from the AES because of their overall profiles (Sec. 2.6).

#### 2.3.3 Minimal Rounds and Security Margins

The two previous sections summarize the known concrete information about the general security of the candidates. Beyond such concrete information lies an array of more heuristic information, much of which is difficult to quantify. Perhaps the most quantifiable dimension (i.e., security margins) is explored in this section; Secs. 2.3.5 and 2.3.6 explore some considerations which are less quantifiable.

Many different phases of an algorithm are subject to attack. The resistance of a particular phase against attack is generally difficult to quantify; an example is key schedule strength. On the other hand, some notable attacks (e.g., differential and linear cryptanalysis) are directly round-oriented. That is, such attacks may be effectively resisted if the number of rounds is high enough. This suggests a quantitative (though obviously incomplete) measure of the security of a candidate (i.e., the actual number of rounds vs. the minimal number needed for security). Unfortunately, the latter is difficult to measure in any absolute sense; the best that can be hoped for is an estimate based on the current state of the art in cryptanalysis.

A scheme for measuring the minimal number of rounds needed for a candidate to be secure against known attacks was formally introduced in Ref. [5]. The precise method used to obtain this minimal number has been the subject of some controversy. A weakness of the treatment in Ref. [5] is that “educated guesses” are freely intermixed with concrete information. In addition, a technical detail of the scheme of [5], namely the automatic addition of 2 rounds (or the equivalent) to each raw minimal number, has been received with some skepticism (e.g., [37]).

An alternative measurement scheme is to rely exclusively upon the greatest number of rounds at which an attack currently exists, and to use this raw number *per se.* Neither approach is correct or incorrect in any absolute sense. The significance of any such measure should be carefully considered; the bottom line is that all such measurements are estimates, regardless of whether a particular estimate is closer to a guess or to something concrete.

If the validity of any such measurement scheme is assumed, it leads to the corresponding notion of *security margin.* For example, if the minimal number of rounds needed for a candidate to be secure is measured as 10, and the actual number of rounds specified is 12, then the security margin is measured as 20 %. Table 5 in the Appendix gives security margins for the candidates, using two measurement schemes (i.e., that of [5] and the alternative measurement scheme based on raw numbers). However, the two schemes are not disjoint; many of the alternative measurements constitute a reformulation of the information in Ref. [5]. Note: NIST does not vouch for the accuracy of any data quoted in this report, if said data is obtained from a source outside NIST.

In constructing profiles of the candidates, NIST gave some consideration to security margins; however, it was kept in mind that such measures are at best approximate, and are based exclusively on defense against existing attacks, as described in public analysis.

#### 2.3.4 Provable Security Claims

In Ref. [17], NIST called for the submissions to include analysis of the security of the algorithms. One candidate, DFC, offered a form of evidence called “provable” security. This does not refer to an absolute proof of security, but rather to proofs that the “decorrelation module” in the round function makes DFC secure against some forms of attack, under a certain mathematical model.

The value of provable security is called into question by [26], in which differential attacks outside the framework of the model are applied to decorrelated ciphers in general and reduced versions of DFC in particular. The submitters of DFC acknowledge in Ref. [4] that a decorrelation design “has to be heuristically secure without the decorrelation modules,” and that “provable security is not a panacea outside its domain of applicability.” Nevertheless, the submitters maintain that “decorrelation properties provide an additional level of security,” and that provable security “provides added value to new designs.”

#### 2.3.5 Design Paradigms, Ancestry and Prior Art

One method of classifying the candidates is by *design paradigm*, although the methodology for doing so is not completely standard. In Ref. [5], the candidates are classified as follows:

- Feistel: DEAL, DFC, E2, LOKI97, MAGENTA, RC6, Twofish.
- Extended Feistel: CAST-256, MARS.
- Substitution Permutation: SAFER+, Serpent.
- Square: CRYPTON, Rijndael.
- Other: FROG, HPC.

A slightly different classification is given in Ref. [22]. However, the details do not seem to matter very much. The question arises as to whether there is a correlation between the design paradigm chosen and the security of the resulting cipher. The answer is not clear. For example, one of the oldest and best-understood design paradigms is Feistel, but three of the seven Feistel candidates (per the above grouping) have apparently had major security gaps exposed (cf. Sec. 2.3.1). At the opposite extreme, the candidates employing nonstandard design paradigms (FROG and HPC) have also had apparent weaknesses exposed. Thus, it appears to be difficult to draw any reliable conclusions from paradigm alone.

A second consideration is ancestry. Some of the candidates are descendants of a relatively long line of members of a family. For example, RC6 is descended from RC5, which has been subject to prior analysis. Similarly, LOKI97 is the latest version of a family that dates to 1989. However, LOKI97 apparently has a major security gap (cf. Sec. 2.3.1). Thus it appears that lineage alone does not guarantee security, although it may provide some insight into candidates.

A review of the public comments received by NIST indicates that the reputation and prior art of the designers of the candidates provided the public with more confidence in the security of the candidate algorithms than the membership of the candidates in any particular family or category.

#### 2.3.6 Simplicity, Cleanness, and Confidence

An even less tangible criterion that could be applied to the candidates is simplicity. This would be difficult to quantify; however, it appears to have played a role in influencing the confidence of the AES community in the security of the candidates. Some candidates (notably, RC6) were praised for simplicity of design; others were criticized for complexity of design. It should be carefully noted that simplicity is not equitable to security. The only assertion that appears to have some real substance is that a simple design may be more amenable to analysis than a more complex one, especially in a restricted timeframe.

Another intangible criterion is cleanness, as defined in Ref. [27]. This refers to a very specific issue (i.e., whether a candidate mixes group operations such as XOR and addition). Although this criterion is more concrete than general simplicity, it is not clear whether “cleanness” can be made any more rigorous in terms of implications for security.

### 2.4 Efficiency

This section focuses on efficiency with respect to general-purpose computer platforms. It largely omits issues specifically pertaining to smart cards; these are treated separately in Sec. 2.5.

#### 2.4.1 Platforms

There has been considerable debate within the AES community as to which platforms are most relevant in regard to measuring the efficiency of candidates. Some people have commented that the original NIST platform specification was not comprehensive enough. This concern has been mitigated somewhat via the efforts of the AES community; the candidates have been implemented on a variety of platforms, and relative efficiency data on many platforms is available. In addition, many opinions have been expressed regarding the relative merits of these platforms. The question of how to measure candidates across platforms is an issue that will undoubtedly continue to warrant discussion.

##### 2.4.1.1 Machine Word Size

One issue that arises is basic underlying architectures. The NIST platforms were exclusively oriented to 32 bit architectures. However, performance on 8 bit and 64 bit machines is also important, as was recognized in the public comments and analyses. It is difficult to project how various architectures will be distributed over the next 10 to 30 years; hence, it is difficult to assign weights to the corresponding performance figures. Nonetheless, from the information received by NIST, the following picture emerges:

It appears that over the next 10 to 30 years, 8 bit, 32 bit, and 64 bit architectures will all play a significant role (in fact, 128 bit architectures will presumably be added to the list at some point). Although 8 bit architectures used in certain applications such as smart cards will gradually be supplanted by 32 bit versions, 8 bit architectures will not disappear. 8 bit architectures will still be used in some low-end smart cards and in similar cryptographic modules. Meanwhile, some 32 bit architectures will be supplanted by 64 bit versions at the high-end; but 32 bit architectures will become increasingly relevant in low-end applications, so that their overall significance will remain high. Meanwhile, 64 bit architectures will grow in importance. Since none of these predictions can be quantified, it appears that versatility is of the essence (i.e., an AES should exhibit good performance across a variety of architectures). Some candidates were specifically oriented to a particular word size (e.g., 8 bits or 64 bits) and did not port well to other architectures. This was taken into account by NIST when seeking Round 2 candidates.

##### 2.4.1.2 Other Architectural Issues

Besides word size, various features of architectures affect performance of the candidates. For example, some architectures provide better support for variable rotations than others. The same is true for multiplications. In addition, 32 bit operations of this sort may be difficult to port to 8 bit or 64 bit machines. It is more difficult to characterize the performance of candidates that require special architectural support than for candidates that rely on operations (e.g., table lookups or XORs) which adapt easily to various architectures. Candidates requiring special architectural support produce performance charts characterized by upward or downward spikes, depending on the presence or lack of appropriate support; the latter produce smoother charts. This does not mean that an algorithm benefiting from special support is inferior or superior to the more uniform performers. However, this factor needs to be taken into account when attempting to select an AES algorithm(s) that will perform well on various platforms.

##### 2.4.1.3 Software

The performance of candidates also depends to some degree on the particular software used (e.g., assembler, compiler or interpreter). In some cases, the role played by particular software has a strong effect on performance figures. For example, optimized, hand-coded assembly code will generally produce better performance than even an optimizing compiler. In addition, some compilers do a better job than others in making use of the support provided by the underlying architecture for operations such as 32 bit rotations. Again, this increases the difficulty of measuring performance across a variety of platforms.

There is no clear consensus on the relative importance of different types of software. In Ref. [37], the opinion is expressed that assembler is the best means of evaluating performance on a given architecture. The reason provided is that hand-coded assembler will be employed in situations in which speed is of the essence and hardware is not available. However, from the rest of the information received by NIST, there does not appear to be universal agreement with respect to this viewpoint.

#### 2.4.2 Measured Speed on General Platforms

An enormous amount of data has been gathered on speeds of candidates on various platforms. The majority of this data pertains to 32 bit platforms, which, as noted earlier, are not necessarily indicative of the environments in which the majority of AES applications over the next 20 years will be implemented. A lesser amount of data has been accumulated for 64 bit architectures, but not much detailed information on 8 bit and/or hardware performance is available at this point (hopefully, Round 2 will shed more light on the latter platforms). Table 1 in the Appendix summarizes some information received from the public on speeds; Table 2 reformulates Table 1 by giving relative ranks. NIST has also accumulated information on speeds by testing the ANSI C and Java supplied by the algorithm submitters [33] and [34]. A rough summary of all available information (which pertains to encryption of 128 bit blocks using 128 bit keys) is as follows:

- Uniformly fast: Rijndael, Twofish. These exhibited excellent performance across a variety of platforms.
- Platform-dependent: MARS, RC6. These performed well on platforms providing a high degree of support for 32 bit multiplications and variable rotations, but not as well otherwise.
- Word size-dependent: DFC, HPC. These are oriented to 64 bit architectures, and do not port well to lower word sizes.
- Mediocre: CAST-256, CRYPTON, E2, and Serpent. These algorithms performed fairly uniformly across platforms, but at slower speeds than Rijndael or Twofish.
- Slow: DEAL, FROG, LOKI97, MAGENTA, SAFER+. These were slow across most platforms.

As discussed earlier, it is difficult to interpret speed data, or groupings of candidates such as the above, in a totally ordered framework; partial ordering is the best that can be hoped for. Thus, in some cases it is possible to say that one algorithm is faster than another, if this holds in a uniform sense; but this type of linear comparison may not hold. Also, there is a segment of the AES community that regards speed data as strictly secondary, and places far more emphasis on security. NIST agrees that speed is not the primary determining factor in assessing a candidate; thus, one slower algorithm (Serpent) was promoted to Round 2, ahead of some faster candidates.

#### 2.4.3 Fair Speed

The notion of “fair speed” is introduced in Ref. [5]. The idea is to utilize some measure of the minimal number of rounds for a candidate, as discussed in Sec. 2.3.3. Then, a hypothetical version of a candidate is constructed which uses this minimal number of rounds. Speeds for these hypothetical versions are then compared.

NIST does not endorse the notion of fair speed comparisons, because they deal with hypothetical versions of the algorithms as opposed to the submitted versions. As noted in Sec. 2.3.3, some “fair speeds” are really guesses, as are the underlying estimates of minimal number of rounds for security. On the other hand, [5] notes that in the process of constructing candidates, different authors made different decisions on the issue of speed/security tradeoff. NIST agrees that such tradeoffs were undoubtedly made, and that the candidates should be neither rewarded nor penalized based purely on their decision for handling the security/speed tradeoff. Rather, it was decided that both security and speed should be taken into account. NIST considered a wide spectrum of speed/security tradeoffs to be *a priori* viable, as long as some security margin was provided according to the “raw” measurement scheme discussed in Sec. 2.3.3.

Some observers have criticized some of the candidates for having “inadequate” security margins. Suggestions have been made (e.g., [27]) regarding possible changes to the number of rounds of some candidates, in order to increase security margins. Each submitter had the opportunity to propose changes to their candidate algorithm prior to Round 2; however, no modifications were proposed for changing the number of rounds for any candidate.

#### 2.4.4 Memory Usage

Another way to examine efficiency is via memory usage. Candidates that use large quantities of memory during execution can cause problems in memory-restricted environments (e.g., the candidate cannot operate in the environment at all). One motivation for the use of Java as a reference platform was to obtain some idea of the dynamic memory usage of candidates. Obtaining such information can be more problematic in some other programming environments. Some results for Java are summarized in Table 9; see also [2].

#### 2.4.5 Encryption vs Decryption

In the cases of some candidates, encryption and decryption utilize identical functions, except for the reversal of the key schedule. In other cases, encryption and decryption are distinct functions. This has some impact on the measurement of efficiency. For example, Table 1 in the Appendix is based on encryption speed. However, some candidates have different speeds for encryption and decryption. These cannot simply be averaged, since there are many applications that require only encryption or decryption, but not both. Most candidates do not exhibit a significant performance decrease during decryption. An exception is FROG, whose decryption function is about half as fast as encryption. For all other candidates, decryption speed is generally no more than about 6 % slower than encryption speed, although there may be specific environments in which a greater discrepancy occurs.

Another consideration is the extra space needed for decryption, in the event that the decryption function is different from the encryption function and both must be included in an implementation. The penalty in this regard depends on the amount of shared code between the two functions. In addition, the significance of this penalty depends on how significant space requirements are and upon the total amount of space needed to house both functions. Note that in some environments, such as certain restricted memory applications or certain modes of operation, it may be necessary to implement only one function, so that there is no general space penalty associated with separate encryption and decryption functions.

#### 2.4.6 Key Computation Options

There are two basic modes for key schedule computation: precomputation and storage of subkeys, and on-the-fly computation of the latter (i.e., computation of one subkey at a time). Some candidates support the on-the-fly option; others do not. This is an oversimplification; there are schemes for subkey computation that lie between precomputation of all subkeys and computation of one subkey at a time (cf. Sec. 2.8.3).

In the case of candidates supporting on-the-fly sub-key computation, a second consideration may arise. If the decryption function requires the subkeys in the opposite order from the encryption function, a candidate may require the key schedule to be computed in the forward direction and then “unwound” [37]. On the other hand, some candidates permit subkeys to be computed in any order. This issue does not arise if the encryption and decryption functions use separate key schedules.

Based on these considerations, the candidates may be classified as follows:

- No direct support for on-the-fly subkey computation: E2, FROG, HPC, LOKI97, MARS, and RC6.
- Support for on-the-fly subkey generation, with:
  - Separate key schedules for encryption and decryption: CRYPTON, Rijndael.
  - Subkeys computable in any order: MAGENTA, SAFER+, and Twofish.
  - Subkeys computable in the forward direction only and requiring unwinding for decryption: CAST-256, DEAL, DFC, and Serpent.

Although this issue is discussed in this section because it is a general property of candidates, different modes of key computation are mainly relevant in restricted-memory environments (cf. Sec. 2.5).

#### 2.4.7 Other Versatility and Flexibility

The previous section discussed one example of the versatility of some candidates; however, there are various other possibilities. Since there are many possible features in this regard, no attempt will be made to catalog these. One common example deals with table representation and/or computation: if a candidate uses large tables, there may be an option for on-the-fly table computation. This may be relevant in adjusting space/time tradeoff decisions to particular environments. Another example is treated separately in the next section.

Some candidates also exhibited some degree of flexibility in the treatment of parameters such as key and round sizes. For example, both MARS and RC6 support key sizes much larger than the 256 bits required in the AES (unfortunately, in both cases there are weaknesses associated to very large key sizes; e.g., 1248 bit keys). In addition, some candidates offer some support for interpolated sizes (e.g., from 128 bit to 256 bit keys in increments of 32 bits). Furthermore, in some cases round numbers were parameterized (e.g., RC6).

Versatility and flexibility played a tertiary role in assessing candidates in Round 1. Such tertiary considerations could conceivably play a greater role in Round 2.

#### 2.4.8 Key Agility

In cases where large amounts of data are processed with a single master key, the setup time for the key schedule may be unimportant, unless it is enormous. On the other hand, in applications in which the key is changed frequently, key agility is a factor. One basic component of key agility is key setup time; some measurements of key setup times are given in Table 3 in the Appendix (see also [2]). A rough summary of this data, accumulated on only a limited number of platforms, is as follows:

- Slow: FROG, HPC. These generally take over 100 000 clock cycles for a 128 bit key setup.
- Medium: CAST-256, DEAL, DFC, E2, LOKI97, MARS, SAFER+, Serpent, and Twofish. These generally take from 2000^2^ to 10 000 clock cycles for a 128 bit key setup.
- Fast: CRYPTON, MAGENTA, RC6, and Rijndael. These generally take less than 2000 clock cycles for a 128 bit key setup.

There are other factors that enter into key agility as well. Such factors tend to arise in restricted-memory environments such as smart cards, and are noted in Sec. 2.5.

#### 2.4.9 Variation of Speed with Key Length

The major key length considered when measuring speed during Round 1 was 128 bits. However, a few candidates have encryption speeds that vary with key length because of an increase in the number of rounds with increasing key length. For example, Rijndael is 20 % slower for 192 bit keys than for 128 bit keys, and 40 % slower for 256 bit keys. Similar variations exist in DEAL, MAGENTA, and SAFER+. These speed decreases are entirely in line with expectations (i.e., extra security gained by increasing the number of rounds is traded off against speed loss).

#### 2.4.10 Potential for Parallelism/Optimal Theoretical Performance

It is to be anticipated that future processors will support various modes of parallelism to a greater extent than existing processors do. This raises the following type of question: if an unlimited amount of processing power is available, so that any potential parallelism in a candidate can theoretically be exploited, to what extent can the candidate take advantage of this situation? The answer to this question is necessarily vague, since explorations can only be carried out on hypothetical machines. Nonetheless, some information can be gleaned from an examination of the operations to be executed for a candidate. One concept in this regard is that of a critical path through code [11]: each instruction can be weighted according to the number of latent clock cycles. A critical path could then be defined to be the path from plaintext to ciphertext with the highest weight. Such measurements are only approximate, and only a small amount of information is available in this regard at present. Some information about theoretical support for parallelism may be found in Table 7 in the Appendix; unfortunately, it applies to only the subset of the candidates (which were studied in Ref. [11]). Therefore, this information did not play a role in deciding which candidates advanced to Round 2. Further public study of parallelism during Round 2 would be welcomed by NIST, and the information in Ref. [11] may be a valuable starting point for that study.

A closely related concept is that of optimal theoretical performance [21]. This measure also depends on a notion of critical path. The conclusions of [21] are summarized in Table 7 in the Appendix.

### 2.5 Smart Card Implementations

A number of security and efficiency issues arise in smart card and related environments; as noted earlier, these were largely deferred to this section.

#### 2.5.1 Low-End Suitability

There is no standard delineation of low-end and high-end smart cards. For the purpose of this discussion, a low-end card will refer to a card with about 256 bytes of RAM available. In theory, intermediate forms of storage such as EEPROM could be used for non-static quantities such as keys. In practice, however, this would be impractical in many instances, since it would negatively impact key agility. Thus, in particular, it must be assumed that subkeys are stored in RAM. In addition, even if a card has 256 bytes of RAM available, it cannot be assumed that a large portion of this space is available for subkey storage. A more conservative (and probably more realistic) assumption is that only about 128 bytes of RAM can be used for this purpose. Also, ROM consumption should be reasonable; it is generally assumed in this report that 2000 bytes of ROM meets this criterion, although some low-end cards may support more.

The issue thus arises as to whether a candidate can be implemented on low-end cards. A major advantage (and in most cases, a virtual prerequisite) is support for on-the-fly key generation (or some equivalent scheme which obviates the necessity of computing and storing all subkeys in advance), as discussed in Sec. 2.4.6. In addition, ROM usage must be reasonable. The only systematic accumulation of RAM and ROM information made available to NIST is in Ref. [37]; Table 4 in the Appendix summarizes this information. Roughly, the situation is as follows:

- CRYPTON, DEAL, DFC, MAGENTA, Rijndael, SAFER+, Serpent, and Twofish are implementable on cards having at least 256 bytes of RAM and 2000 bytes of ROM.
- CAST-256, E2, MARS, and RC6 require more than 256 bytes of RAM, 2000 bytes of ROM, or both. However, they are suitable for higher-end cards (e.g., 512 bytes of RAM and 6000 bytes of ROM would accommodate each of these four candidates).
- FROG, HPC, and LOKI97 are poorly suited to smart cards, needing more than 2000 bytes of RAM, 6000 bytes of ROM, or both.

As noted earlier, the above classifications are heavily dependent on the precise storage limits used as delineators. Also, there is some disagreement as to how important implementability on low-end cards is to the AES. This issue was not directly confronted in Round 1; two candidates unsuitable for low-end cards (referring to the original submissions) were promoted to Round 2. However, the proposed modification for MARS is claimed by the submitter to address that deficiency. Since the other three Round 2 candidates are suitable for implementation on low-end cards, this issue is sure to arise in the future.

#### 2.5.2 Vulnerability of Operations to Timing and Power Attacks

Timing attacks can be effected against operations that execute in different amounts of time, depending on their arguments. Power analysis attacks can be effected against operations that use different amounts of power, depending on their power consumption pattern, which may vary with the arguments to the operation. A possible defense against power analysis attack is software balancing. This technique may be effective for certain operations whose power consumption can be “masked” to some extent by executing the operation twice, employing the complement of argument(s) the second time. A rough summary of vulnerabilities of the operations used in candidates is as follows [12]:

- Table lookup: not vulnerable to timing attacks. Defense against power attacks may be effected by using the address and its complement.
- Fixed shifts/rotations: not vulnerable to timing attacks. Defense against power attacks may be effected by using the contents of the register containing the shift/rotate amount, and its complement.
- Boolean operations: not vulnerable to timing attacks. Defense against power attacks may be effected by using the complements of arguments.
- Addition/subtraction: somewhat difficult to defend against timing or power attacks.
- Multiplication/division/squaring or variable shift/rotation: most difficult to defend against timing and power attacks.

Note: in the above, saying that a defense may be effected does not guarantee that a given operation is in fact protected; it merely means that such a defense is theoretically possible. Conversely, saying that an operation is difficult to defend against an attack does not imply that any given implementation of a candidate employing that operation is vulnerable to attack. A discussion of the actual attacks and defenses against them is outside the scope of this paper.

A summary of the operations used by the candidates is given in Table 6 in the Appendix. A rough summary of this information is as follows:

- CRYPTON, DEAL, MAGENTA, Rijndael, and Serpent use only Boolean operations, table lookups, and fixed shifts/rotations. These are easiest to defend against attacks.
- FROG, LOKI97, SAFER+, and Twofish use addition/subtraction, and are somewhat more difficult to defend against attacks.
- CAST-256, DFC, E2, HPC, MARS, and RC6 use multiplication/division/squaring and/or variable shift/rotation, and are most difficult to defend.

Note: saying that a candidate uses an operation which is difficult to defend does not mean that the candidate is indefensible; this simply refers to the complexity of the task of defense. For example, in some cases, software defenses may be sufficient to defend against a particular attack. In other cases, this may be infeasible, and some form of hardware defense may be necessary. Furthermore, there is no guarantee that in a particular situation, any form of defense will work. That is, timing and power analysis are implementation-dependent attacks; the vulnerability of candidates to such attacks is not an intrinsic algorithm characteristic. Thus, considerations such as the above did not play a primary role in deciding advancement to Round 2; much more attention was paid to general attacks which are essentially implementation-independent.

#### 2.5.3 Implicit Key Schedule Weaknesses

In Ref. [9] an interesting theory is developed concerning the resistance of the key schedules of the candidates to attack. A more general question is the following: if an attacker gains access to a subkey (or, in some cases, a whitening key), does this yield information about other subkeys or the master key? If so, this might be termed an implicit (or perhaps conditional) key schedule weakness. Unfortunately, the classification of key schedules in Ref. [9] is somewhat ill posed. Nonetheless, this theory raises an issue that has had significant consequences in practice, particularly in connection with attacks on smart card implementations. Further research on this subject would be useful. At the present time, two attacks are known which exploit implicit key schedule weaknesses; these are discussed below. NIST has not verified these attacks. Hence, implicit key schedule weaknesses did not play a significant role in determining advancement to Round 2. These attacks, and defenses against them, require further study.

##### 2.5.3.1 A Power Analysis Variant

In Ref. [6], the authors employ a variant of power analysis to attack the key schedules of the candidates when implemented in smart cards. Their approach correlates power consumed with the number of ones in a byte of a subkey. Evaluating the number of ones yields an equation involving the bits of the master key, regarded as independent variables. A sufficient number of such evaluations may provide a system of equations that can be used to obtain the master key, assuming a sufficiently high rank. The rank in turn depends upon the randomness of the process used to generate subkeys from the master key; conversely, redundancy in this process inhibits the attack by lowering the rank. However, even if the full master key cannot be recovered, it may still be possible to obtain some information about it.

Assuming that such a power analysis attack can in fact be effected, a rough classification of candidates by key schedule is as follows:

- Significant implicit weaknesses: CAST-256, CRYPTON, DEAL, DFC, E2, and SAFER+. These are highly vulnerable to the attack (the equivalent of at least 50 % of the master key can be recovered).
- Lesser implicit weaknesses: LOKI97, MARS, RC6, and Rijndael. Attack may reveal some information about the master key.
- No weakness: MAGENTA, Serpent, and Twofish.
- FROG, HPC: these are not well suited to smart card or similar applications in which key schedule weaknesses are typically exploited.

Note: [6] should be consulted for the precise conditions under which the above attack can be executed. Saying that a candidate is vulnerable to the attack presupposes that the attack is in fact feasible for a given implementation of a candidate. As in Sec. 2.5.2, vulnerability to this type of attack is not an intrinsic algorithm characteristic. Thus, saying that a candidate has an implicit weakness that might be exploited under certain conditions simply means that certain defenses may be needed to defend against the attack. There might also be restrictions on the class of smart cards for which a given candidate is suitable. More generally, the feasibility of an attack such as the above may be impacted by algorithm characteristics, implementation characteristics, card characteristics, and usage scenarios. A discussion of the feasibility of such an attack, and defenses against it, is outside the scope of this paper.

##### 2.5.3.2 Another Power Analysis Variant

In Ref. [10], yet another variant of power analysis is employed to attack the key schedules of candidates when implemented in smart cards. The attack exploits the particular operations used to generate subkeys. If some subkeys (or, in some cases, whitening keys) can be found, it may be possible to obtain information about other subkeys or the master key. Viability of the attack depends partially on the number of rounds that need to be attacked to obtain the sought-after information. From the point of view of this attack, the candidates may be classified roughly as follows:

- Most vulnerable: CRYPTON, DEAL, LOKI97, MAGENTA, Rijndael, SAFER+, Serpent, and Twofish. Their vulnerability springs from the derivability of the master key from a small number of subkeys or whitening keys. Only a small number of rounds need to be attacked.
- Less vulnerable: CAST-256, DFC, E2, MARS, and RC6. All or a large number of rounds need to be attacked.
- FROG, HPC: these are not well suited to smart card or similar applications in which key schedule weaknesses are typically exploited.

Note: as in Sec. 2.5.3.1, saying that a candidate is vulnerable to the attack presupposes that the attack is feasible. See [10] for a discussion of the precise conditions under which the attack is feasible. In addition, again it should be noted that vulnerability to such an attack is not an intrinsic algorithm characteristic, but rather is heavily implementation-dependent.

#### 2.5.4 Some Possible Defenses

Various mechanisms have been proposed to defend against timing and power analysis attacks, such as those discussed in Sec. 2.5.3. Proposed defense mechanisms include (e.g., [12]):

- Elimination of branching in program execution, to defend against timing attacks.
- Software balancing (e.g., using complements of arguments to even out the total power consumed).
- Algorithm design (e.g., avoiding operations that are difficult to defend, and avoiding implicit key schedule weaknesses).
- Hardware mechanisms (e.g., random noise production).
- Choice of card.
- Operational defenses.

Notes on the above: software balancing and algorithm design strategies have been discussed earlier. The other mechanisms relate to the fact that the essence of most attacks is to collect statistical samples of quantities such as power consumption. Hardware defenses may raise the noise to signal ratio, requiring the number of samples to be higher.

The choice of card is significant in several respects. First, high-end cards may have hardware defenses unavailable in lower-end cards. Secondly, attacks often model cards as finite-state automata. The difficulty in effecting an attack (reflected in the number of statistical samples of a quantity such as power consumption) may be related to the number of possible states of the card. The number of states determines, in part, the complexity of the state space that must be analyzed. The latter may be greater for high-end cards.

Operational defenses also relate to the sampling phase of an attack. It may be possible to limit an attacker’s ability to obtain samples pertaining to one key (e.g., by limiting the number of encryptions which can be performed by one key). Of course, an obvious method of defending against timing or power attacks is to physically protect the card. This is feasible in cases in which the owner of the card is not a potential adversary of the entity that places keys on cards (in particular, when the card stores only keys generated by the owner).

#### 2.5.5 Performance

It is generally agreed that speed on smart cards is a tertiary consideration, with implementability and security being more fundamental. In addition, a peculiar feature of smart card and related environments is that implementations may need to be protected against attacks such as power analysis. This may mean that the most efficient implementation of a candidate on a given card is undesirable for security reasons. Another consideration is that speed and space may be traded off (e.g., by on-the-fly vs. precomputed generation of data objects). Thus, it is more difficult to assess performance of candidates on smart cards than on general-purpose computers.

For these reasons, concrete information about the performance of candidates on smart cards is sketchy at present. One collection of speed measurements on 8 bit processors is given in Table 8 in the Appendix.

#### 2.5.6 Related Environments

Various other environments share characteristics in common with smart cards. A notable example is hardware implementations. All such environments are characterized by restrictions on RAM and ROM, due either to lack of space or cost that increases linearly with space. Hence, some of the above discussion extrapolates to other environments as well. For example, a candidate with a large RAM requirement will probably be costly in hardware, paralleling the lack of implementability on low-end smart cards.

There is even less concrete (and homogeneous) information available for environments such as hardware than for smart cards. Hopefully, this gap will be addressed in Round 2.

### 2.6 Profiles of the Candidates

The following summarizes the salient information that NIST was able to accrue about the candidates during Round 1. In cases where modified versions have been submitted, profiles are still based largely on the original versions, since modified versions have not been subject to public inspection or thoroughly analyzed by NIST. See Sec. 2.8 for further information on the modification proposals.

Security gaps are divided into two distinct categories. General (major and minor) security gaps refer to those attacks that can be executed in a manner that is essentially implementation-independent (i.e., these attacks exploit intrinsic algorithm weaknesses). In assessing security of candidates, such general attacks were regarded as being the primary area of concern. A second category of attacks is implementation-dependent; the major examples of such attacks are related to smart card implementations. Attacks on smart card implementations may exploit intrinsic algorithm characteristics; nonetheless, they cannot be regarded as being indicative of intrinsic algorithm weaknesses, since any given implementation may or may not be vulnerable (cf. Sec. 2.5.3). Thus, statements in the following to the effect that a candidate is vulnerable to a smart card attack mean that certain defenses may be needed in implementations, or that there may be restrictions on the class of feasible implementations.

#### 2.6.1 CAST-256

Major security gaps: none known.

Minor general security gaps: none known.

Advantages:

- High security margin.
- Supports on-the-fly subkey generation.
- Descended from CAST-128, which has previously been analyzed.

Disadvantages:

- Mediocre speed across platforms.
- Large ROM requirement rules out many low-end smart card implementations and makes hardware implementation expensive.

Security gaps related to smart card implementations:

- Difficult to secure against power-analysis attacks due to the use of variable rotations and additions/subtractions.
- Very vulnerable to the attack in Ref. [6].
- Slightly vulnerable to the attack in Ref. [10].

#### 2.6.2 CRYPTON

Major security gaps: none known.

Minor general security gaps: some key schedule weaknesses, as noted in Sec. 2.3.2.

Advantages:

- Good speed across platforms.
- Implementable on low-end smart cards: supports on-the-fly key generation; reasonable ROM requirement.
- Operations employed are easiest to defend against timing and power analysis attacks on smart card implementations.
- Fast key setup.
- Good support for instruction-level parallelism.
- Supports additional key sizes in increments of 32 bits, to 256 bits; also supports a block size of 512 bits.

Disadvantages: relatively low security margin.

Security gaps related to smart card implementations: very vulnerable to the attacks in Ref. [6] and [10].

#### 2.6.3 DEAL

Major security gaps: see Sec. 2.3.1.

Minor general security gaps: see Sec. 2.3.2.

Advantages:

- Implementable on low-end smart cards: supports on-the-fly key generation; reasonable ROM requirement.
- Operations employed are easiest to defend against timing and power analysis attacks on smart card implementations.

Disadvantages: slow across platforms.

Security gaps related to smart card implementations: very vulnerable to the attack in Ref. [10].

#### 2.6.4 DFC

Major security gaps: none known.

Minor general security gaps: see Sec. 2.3.2.

Advantages:

- Implementable on low-end smart cards: supports on-the-fly key generation; reasonable ROM requirement.
- Provable security is provided against some attacks under a certain mathematical model.
- Very fast on 64 bit platforms.
- Supports arbitrary key sizes to 256 bits.

Disadvantages:

- Security margin appears to be relatively low.
- Operations employed (viz., 64 bit multiplications) do not port well to 8 bit or 32 bit platforms.

Security gaps related to smart card implementations:

- Difficult to defend against timing and power analysis attacks due to the use of multiplications and additions.
- Very vulnerable to the attack in Ref. [6].
- Slightly vulnerable to the attack in Ref. [10].

#### 2.6.5 E2

Major security gaps: none known.

Minor general security gaps: see Sec. 2.3.2.

Advantages: adequate security margin.

Disadvantages:

- Relatively mediocre speed across platforms.
- Lack of on-the-fly subkey generation rules out implementation on many low-end smart cards.
- Very high ROM usage (see Table 9 in the Appendix).

Security gaps related to smart card implementations:

- Difficult to defend against timing and power analysis attacks due to the use of multiplications and divisions.
- Very vulnerable to the attack in Ref. [6].

#### 2.6.6 FROG

Major security gaps: see Sec. 2.3.1.

Minor general security gaps: none known.

Advantages: no significant advantages.

Disadvantages:

- Design paradigm makes analysis difficult in a restricted timeframe.
- Very slow key setup.
- Slow speed across platforms.
- Unsuitable for many smart card implementations due to a high RAM requirement.
- Decryption is about twice as slow as encryption.

#### 2.6.7 HPC

Major security gaps: see Sec. 2.3.1 (modified version (cf. Sec. 2.8.2) purports to correct this problem).

Minor general security gaps: none known.

Advantages:

- Good performance on 64 bit platforms.
- Supports arbitrary block and key sizes.

Disadvantages:

- Design paradigm makes analysis difficult in a restricted timeframe.
- Very slow key setup.
- Does not port well to 8 bit or 32 bit platforms.
- Unsuitable for many smart card implementations due to high RAM requirement.

#### 2.6.8 LOKI97

Major security gaps: see Sec. 2.3.1.

Minor general security gaps: none known.

Advantages: no significant advantages.

Disadvantages:

- Slow across platforms.
- Poorly suited to smart card implementation due to large ROM requirement.

#### 2.6.9 MAGENTA

Major security gaps: see Sec. 2.3.1.

Minor general security gaps: none known.

Advantages: no significant advantages.

Disadvantages: slow across platforms.

#### 2.6.10 MARS

Major security gaps: none known.

Minor general security gaps: none known.

Advantages:

- Large security margin.
- Good performance on 32 bit platforms; excellent performance on platforms providing strong support for 32 bit variable rotations and multiplications.
- Supports key sizes much higher than 256 bits (theoretically up to 1248 bits, but some equivalent keys emerge at the boundary).

Disadvantages:

- Performance drops off on platforms not providing the support needed.
- Original version is not well suited to smart card implementation due to lack of on-the-fly subkey generation and borderline large ROM requirement (modified version (cf. Sec. 2.8.3) apparently corrects RAM problem. Status of ROM problem depends on precise ROM limit of card).
- Complexity makes analysis difficult in a restricted timeframe.

Security gaps related to smart card implementations:

- Difficult to defend against timing and power analysis attacks due to the use of multiplications, variable rotations, and additions.
- Slightly vulnerable to the attacks in Ref. [6] and [10].

#### 2.6.11 RC6

Major security gaps: none known.

Minor general security gaps: none known.

Advantages:

- Fast on 32 bit platforms; very fast on platforms providing strong support for 32 bit variable rotations and multiplications.
- Very simple structure facilitates analysis, especially in a restricted timeframe.
- Evolved from RC5, which has been subjected to prior analysis.
- Fast key setup.
- Supports key sizes much higher than 256 bits (theoretically up to 1248 bits, but some equivalent keys emerge at the boundary).
- Key size, block size, and round number are fully parameterized.

Disadvantages:

- Relatively low security margin.
- Performance drops off on platforms not providing support needed.
- Lack of support for on-the-fly key generation rules out implementation on many low-end smart cards.

Security gaps related to smart card implementations:

- Difficult to defend against timing and power-analysis attacks due to use of squaring and variable rotations.
- Slightly vulnerable to the attacks in Ref. [6] and [10].

#### 2.6.12 Rijndael

Major security gaps: none known.

Minor general security gaps: none known.

Advantages:

- Excellent performance across platforms.
- Good security margin.
- Well-suited to smart cards due to low RAM and ROM requirements.
- Operations employed are easiest to defend against attacks on smart card implementations.
- Fast key setup.
- “Clean” as defined in Ref. [27].
- Good support for instruction-level parallelism.
- Supports other key and block sizes in increments of 32 bits.

Disadvantages: no significant disadvantages.

Security gaps related to smart card implementations:

- Very vulnerable to the attack in Ref. [10].
- Slightly vulnerable to the attack in Ref. [6].

#### 2.6.13 SAFER+

Major security gaps: none known.

Minor general security gaps: see Sec. 2.3.2 (modified version (cf. Sec. 2.8.4) submitted to correct this problem).

Advantages:

- Good security margin.
- Well-suited to smart cards due to low RAM and ROM requirements.
- Supports on-the-fly subkey generation with subkeys computable in any order.

Disadvantages: Very slow across platforms.

Security gaps related to smart card implementations:

- Employs addition, which is somewhat difficult to defend against timing and power attacks.
- Very vulnerable to the attack in Ref. [10].

#### 2.6.14 Serpent

Major security gaps: none known.

Minor general security gaps: none known.

Advantages:

- Large security margin.
- Well-suited to smart cards due to low RAM and ROM requirements.
- Operations employed are easiest to defend against timing and power attacks.
- “Clean” as defined in Ref. [27].
- Bitslice implementations can allow for an efficient, parallel computation of S-boxes.

Disadvantages: slow across platforms.

Security gaps related to smart card implementations: very vulnerable to the attack in Ref. [10].

#### 2.6.15 Twofish

Major security gaps: none known.

Minor general security gaps: none known.

Advantages:

- Large security margin.
- Very fast across platforms.
- Well-suited to smart cards due to low RAM and ROM requirements.
- Supports on-the-fly subkey generation with subkeys computable in any order.
- Good support for instruction-level parallelism.
- Versatile; admits several modes of implementation, allowing for space/time tradeoffs.
- Supports arbitrary key sizes up to 256 bits.

Disadvantages:

- Key-dependent S-boxes complicate analysis.
- Overall complexity of design has drawn some concern.

Security gaps related to smart card implementations:

- Employs addition, which is somewhat difficult to defend against timing and power analysis attacks.
- Very vulnerable to the attack in Ref. [10].

### 2.7 Assessments of the Candidates

NIST stated in Ref. [17] that it intended to “narrow the field of candidates to no more than five candidate algorithms” for Round 2 analysis. Doing so will focus the analysis (particularly cryptanalysis) on a small set of the candidates and maximize the amount of information that can be developed on each of the finalists. NIST also believes that the narrowing of the field is appropriate in order to develop the AES in a reasonable timeframe. During the finalist selection process, NIST maintained its goal of selecting five candidate algorithms representing potentially superior technology that would have a very good chance of being selected for the AES FIPS.

The following are NIST’s overall assessments of the candidates, summarizing the results of Round 1 analysis and stating whether NIST advances a candidate to Round 2. For those algorithms that did not have major security weaknesses, NIST examined advantages and disadvantages. NIST also drew comparisons and contrasts between algorithms with similar profiles in order to determine which candidates were better suited for Round 2 analysis and possible selection for the AES FIPS. The assessments below are not intended to be a comprehensive list and description of the features and properties of the algorithms, which were discussed in the preceding sections of this document and in the public comments and analyses. Those sources should be consulted for the specific Round 1 details of each algorithm.

For those candidates advancing to Round 2, the profiles (see Sec. 2.6) and the assessments below will serve as a starting point for further analysis.

#### 2.7.1 Candidates with Major Security Attacks

Round 1 analysis revealed major security attacks against the following five candidates:

DEAL: Serious questions have been raised about the security of DEAL. Additionally, in terms of performance, it is consistently very slow for encryption and decryption across all platforms used in the Round 1 analysis. DEAL is not advanced to Round 2.

FROG: Serious questions have been raised about the security of FROG, in spite of the fact that its design paradigm makes analysis difficult in a restricted time frame. The algorithm also demonstrates slow encryption and decryption speeds across almost all platforms, with decryption being half as fast as encryption. Concerns about extremely slow key setup and large RAM requirements were also raised during Round 1. Additionally, no significant advantages were identified for FROG. For these reasons, FROG is not advanced to Round 2.

HPC: Serious questions have been raised about HPC (original version) due to a very large number of equivalent keys. Also, HPC’s design is extremely difficult to analyze in a restricted time frame. A modified version of HPC claims to correct the security problem (cf. Sec. 2.8.2). Although HPC appears to perform well on 64 bit platforms, it performs slowly on all other platforms, and has an extremely slow key setup time. High RAM requirements also make HPC unsuitable for many smart card implementations. HPC is not advanced to Round 2.

LOKI97: Serious questions have been raised about the security of LOKI97, with respect to both linear and differential cryptanalysis for relatively few known and chosen plaintexts. This algorithm also exhibits poor performance across the platforms used in the Round 1 analysis. Large ROM requirements result in poor suitability for smart card implementations. The algorithm does not possess any significant, identifiable advantages. LOKI97 is not advanced to Round 2.

MAGENTA: Serious questions have been raised about the security of MAGENTA, with respect to a chosen plaintext attack. Encryption and decryption speeds for MAGENTA are consistently among the slowest of any of the 15 Round 1 candidates. No significant, identifiable advantages of the algorithm were identified during Round 1. MAGENTA is not advanced to Round 2.

#### 2.7.2 Candidates without Major Security Attacks

Below are five algorithms that had either minor or no security weaknesses identified during Round 1. They also possessed varying degrees of advantages and disadvantages.

CAST-256: This candidate belongs to a class of candidates with the speed/security ratio tilted strongly in favor of security. This resulted in inevitable comparisons to Serpent, which has a comparable security profile. In terms of performance, CAST-256 appears to have mediocre speed across most platforms. Another disadvantage of CAST-256 is its relatively high ROM Disadvantages: requirement, which rules out many low-end smart card implementations and increases the expense of hardware implementations. On the other hand, Serpent is more versatile, and its low ROM requirements make it better suited for restricted-memory environments. Thus, the edge in this class appears to belong to Serpent. Since it was considered unlikely that two algorithms with this type of profile would be chosen for the AES FIPS, CAST-256 was deemed to have a low chance of becoming an AES, and, therefore, is not advanced to Round 2.

CRYPTON: This candidate has the same general profile as candidates like Rijndael and Twofish, but CRYPTON has a lower security margin, based on evidence produced during Round 1. Additionally, the original version of CRYPTON has a key schedule weakness (a modified version was submitted but rejected (cf. Sec. 2.8.1)). Rijndael and Twofish have no known general security gaps. Regarding performance, CRYPTON is among the faster of the candidates, but it is slower than either Rijndael or Twofish on most platforms. Taking into account all of these factors, it was considered unlikely that CRYPTON would surpass either Rijndael or Twofish when the AES algorithm(s) is selected. Thus, CRYPTON is not advanced to Round 2.

DFC: The precise security status of DFC is difficult to ascertain due to a lack of resolution of the issue of provable security ([26], [39]). It has been argued that the scope of the submitter’s provable security claims is sufficiently limited such that a case for a strong proof of overall security cannot be made. In addition, there is a concern about DFC’s low security margin. Thus, the only concrete advantage of DFC appears to be its performance on 64 bit platforms. However, DFC’s performance drops off substantially on platforms with smaller word sizes, due to its use of 64 bit multiplications. There are other candidates (e.g., Rijndael) that perform roughly the same on 64 bit platforms and are much more versatile with respect to word size, in addition to having larger security margins. It was deemed unlikely that DFC would be chosen ahead of some of the more versatile candidates. Hence, DFC is not advanced to Round 2.

E2: The general profile of E2 is similar that of Rijndael and Twofish, although E2’s security margin is lower. In terms of performance, E2 is slower than Rijndael and Twofish on most platforms. Due to its high RAM requirement, E2 is not implementable on low-end smart cards, unlike Rijndael and Twofish. A high ROM requirement is also a problem for E2. For these reasons, it was considered unlikely that E2 would surpass either Rijndael or Twofish when the AES algorithm(s) is selected. Thus, E2 is not advanced to Round 2.

SAFER+: Although SAFER+ has a good security margin, another candidate with a similar profile– Serpent– has a significantly higher margin. SAFER+ is very slow, especially on 32 bit and 64 bit platforms, but the lack of speed is not fully commensurate with its security margin (unlike Serpent). Hence, it was felt that if a slow candidate were to be chosen for the AES FIPS, it would more likely be a candidate such as Serpent that exhibits a better performance/security profile. SAFER+ is not advanced to Round 2.

#### 2.7.3 Candidates Selected for Round 2

Based on the Round 1 analysis and comments, the following five candidates had no major or minor security gaps, and possessed numerous advantages:

MARS: One of MARS’ strengths is its large security margin. No security gaps were evident from Round 1 analysis and comments. Additionally, MARS demonstrates very good performance on 32 bit platforms, and its speed excels on platforms that provide strong support for 32 bit variable rotations and multiplications. The algorithm also has the flexibility to handle key sizes much higher than the required 256 bits. Some concerns about MARS include its performance on platforms that do not provide the support needed, as well as the algorithmic complexity. The effect of a heterogeneous round structure on security would be an appropriate subject of further study. Overall, it was felt that the plusses significantly outweighed the minuses; hence, MARS is advanced to Round 2.

RC6: The simplicity of RC6 is very attractive, especially with respect to facilitating security analysis in a restricted timeframe. No security gaps were evident from Round 1 analysis and comments. RC6 is very fast on 32 bit platforms, and it is the fastest performer on any platform providing the needed support. Its key setup time is very fast. Additionally, this algorithm is extremely flexible in the sense that the key size, block size, and number of rounds are all fully parameterized. Some concerns about RC6 include the relatively low security margin, declining performance on platforms that do not provide strong support for 32 bit variable rotations and multiplications, and the lack of low-end smart card suitability. Overall, it was felt that the plusses significantly outweighed the minuses; hence, RC6 is advanced to Round 2.

Rijndael: Rijndael is an excellent candidate on all major counts, Rijndael has had no significant disadvantages identified. During Round 1, no major or minor security gaps were evident, and the algorithm appears to have a good security margin. Fast key setup, and consistently excellent performance across all platforms considered during Round 1 are very positive characteristics. The algorithm’s low RAM and ROM requirements also make it very suitable for smart cards. The ability to handle additional key and block sizes also contributes to the algorithm’s outstanding flexibility. Rijndael is advanced to Round 2.

Serpent: Serpent has one of the strongest overall security profiles and is extremely well suited for smart cards. It is designed to allow bitslice implementations, in which the S-boxes can be computed by logical operations rather than table lookups. Such optimized versions should allow relatively efficient parallel computation of the S-boxes, especially on 32 bit platforms. Nevertheless, its slow speed remains a minus. Overall, it was felt that the plusses predominate. Hence, Serpent was advanced to Round 2.

Twofish: On almost all major counts, Twofish is an excellent candidate. It possesses a large security margin, and, during Round 1, no major or minor security gaps were evident. In terms of performance, Twofish is very fast across almost all platforms. Low RAM and ROM requirements make it suitable for smart cards and other restricted memory environments. Another advantage is Twofish’s flexibility, since it admits several modes of implementation to accommodate various space/time tradeoffs. During Round 1, there were a few concerns regarding the overall complexity of its design. Twofish is advanced to Round 2.

### 2.8 Modified Versions

NIST’s call for candidate algorithms [17] made an allowance for submitters to propose modified versions of their candidates prior to Round 2. NIST specifically included this in Ref. [17] in case none of the algorithms completed Round 1 without security problems. Since NIST in fact received 21 algorithm submissions, and 15 were included for Round 1, that situation did not occur.

In Ref. [17], NIST stated that “small modifications to the submitted algorithms will be permitted for either security or efficiency purposes…[and] no substantial redesigns” would be allowed. At AES2, NIST expressed its concern that substantial changes would likely invalidate the majority of Round 1 analysis for an algorithm and would, therefore, not be allowed. Additionally, NIST stated that “The security provided by an algorithm is the most important factor in the evaluation” [17]. The submission of a proposed modification to fix a security deficiency was taken by NIST as a recognition that the algorithm should be improved. Since many other algorithms did not have security deficiencies identified during Round 1 (and thus did not propose a security enhancement), NIST viewed the algorithms not requiring security modifications more favorably. If security weaknesses were exposed in an algorithm during Round 1, then NIST was concerned that this might indicate other security problems with the algorithm.

Submitters of four algorithms—CRYPTON, HPC, MARS, and SAFER+—proposed modifications to their original submissions after the close of Round 1. Three of the modification proposals—for CRYPTON, HPC, and SAFER+—are intended to correct a specific security weakness. The modification proposed for MARS addresses efficiency concerns. These modifications are discussed in the following sections.

#### 2.8.1 CRYPTON

Version 1.0 of CRYPTON was submitted as a modification [28] to the original submission. The submitter claims that the modification will correct the key schedule weaknesses of the original submission. However, the modification also changes the S-boxes. It was felt by NIST that the combination of these two changes violates the criterion that a modification should not invalidate the majority of Round 1 analysis. Since changes to both the key schedule and S-boxes would presumably necessitate significant re-analysis, the modification was not accepted.

#### 2.8.2 HPC

The submitter of HPC proposed a modified version [38] that is intended to correct the equivalent-key problem in the original submission. NIST could not ascertain that the modified version is secure. However, even if this modification were successful in correcting the original key schedule problem, it would not alter the overall profile of HPC—particularly regarding performance characteristics—significantly enough to gain admission to Round 2. Thus, the acceptance of this proposal is a moot point.

#### 2.8.3 MARS

A modified version of MARS [45] changes the mode of subkey generation. The submitter claims that the subkeys are now computed in four separate phases, each encompassing a quarter of the subkeys (assuming this description is accurate, the scheme would lie somewhere between precomputed and on-the-fly generation).

While some Round 1 analysis of the algorithm would be invalidated by the proposed modification, it would appear that the limited scope of this change meets the criterion of not invalidating the majority of Round 1 analysis. Hence, this modification was accepted by NIST. The consensus was that this modification would improve a candidate algorithm whose original version was already strong, although the precise extent of this improvement remains to be seen. MARS was originally limited with respect to restricted-memory environments because of both RAM and ROM requirements. The adjustments to subkey generation appear to have reduced the RAM requirement to the point where implementation on low-end cards is feasible. However, ROM requirements remain a concern.

#### 2.8.4 SAFER+

A modified version of SAFER+ [42] was proposed to correct the minor key schedule deficiency noted in Sec. 2.3.2. However, even if this modification produces a secure key schedule, it would not alter the profile of SAFER+ sufficiently to gain admission to Round 2, and thus the acceptance of this proposal is a moot point.

### 2.9 Conclusion

As a result of the analysis of all comments, papers, verbal comments at conferences, NIST studies, reports and proposed modifications, NIST has selected five finalists for AES Round 2: MARS, RC6, Rijndael, Serpent, and Twofish. No significant security vulnerabilities were found for these candidates during the Round 1 analysis, and each of these algorithms constitutes potentially superior technology.

## Figures and Tables

**Table 1:** Encryption speeds of the candidates

	32 bit C and assembler						64 bit C and assembler				Other		
	A	B	C	D	E	F	G	H	I	J	K	L	M
CAST	668	2088	660	600	600	633	749	694	600	615	1275	5160	10733
CRYP	390	1282	476	345	390	474	499	477	408	353	865	3630	4155
DEAL	2339	8950	2600	2200	2200	2339	2752	2781	2528	2010	3940	21800	37991
DFC	480	5874	1700	750		1642	323	802	304	232	2435	367000	22668
E2	415	1808	720	410	410	687	587	711	471	510	990	2310	9652
FROG	2572	2182	2600			2417	2752	2337		3750	2620	8800	
HPC	1468	2602	1600			1429	402	450	376	420	1315	3620	21759
LOKI		6376	2150			2134	2356					4500	
MAG		23186	6600			6539	5074					56200	52681
MARS	376	1600	390	320	550	369	507	840	478	450	950	2960	8908
RC6	243	1436	260	250	700	270	559	1161	467	382	1085	1870	8231
RIJN	284	1276	440	291	320	374	490	328	340	285	735	2230	3518
SAFR	775	4424	1400	800	1100	1722	1502	3002	656	929	5085	8360	10288
SERP	992	1800	1030	900	1100	952	998	992	915	855	1345	3180	14703
TWOF	258	1254	400	258	290	376	490	487	360	315	755	5280	4672

Column headings:

A: “Best-known implementation,” Intel Pentium 2. Source: [29], Table 2.

B: Linux/GCC-2.7.2.2/Pentium 133 MHz MMX. Source: [5], Table 3.

C: Intel Pentium Pro C. Source: [37], Table 2.

D: Intel Pentium Pro ASM. Source: [37], Table 2.

E: Intel Pentium ASM. Source: [37], Table 2.

F: Intel Pentium Pro/II, C. Source: [20], Table 1.

G: Compaq Alpha 21164a 500 MHz, DEC CC. Source: [4], Table 1.

H: “Best-known implementation,” SUNTM UltraSPARCTM. Source: [29], Table 2.

I: DEC Alpha ASM. Source: [37], Table 7.

J: DEC Alpha AXP 21264, DEC CC. Source: [29], Table 1.

K: Hewlett-Packard PA7000. Source: [29], Table 1.

L: Pentium Pro, Java. Source: [35].

M: Texas Instruments TMS320c541 DSP, C. Source: [43].

**Table 2:** Encryption speeds, ranked

	A	B	C	D	E	F	G	H	I	J	K	L	M
CAST	8	8	6	7	6	6	9	5	9	9	7	9	8
CRYP	5	3	5	5	3	5	5	3	5	4	3	7	2
DEAL	12	14	13	11	10	13	13	12	12	12	12	13	12
DFC	7	12	11	8		10	1	7	1	1	10	15	11
E2	6	7	7	6	4	7	8	6	7	8	5	3	6
FROG	13	9	13			14	3	11		13	11	12	
HPC	11	10	10			9	2	2	4	6	8	6	10
LOKI		13	12			12	12					8	
MAG		15	15			15	15					14	13
MARS	4	5	2	4	5	2	6	8	8	7	4	4	5
RC6	1	4	1	1	7	1	7	10	6	5	6	1	4
RIJN	3	2	4	3	2	3	3	1	2	2	1	2	1
SAFR	9	11	9	9	8	11	11	13	10	11	13	11	7
SERP	10	6	8	10	8	8	10	9	11	10	9	5	9
TWOF	2	1	3	2	1	4	3	4	3	3	2	10	3

Column headings:

A: “Best-known implementation,” Intel Pentium 2. Source: [29], Table 2.

B: Linux/GCC-2.7.2.2/Pentium 133 MHz MMX. Source: [5], Table 3.

C: Intel Pentium Pro C. Source: [37], Table 2.

D: Intel Pentium Pro ASM. Source: [37], Table 2.

E: Intel Pentium ASM. Source: [37], Table 2.

F: Intel Pentium Pro/II, C. Source: [20], Table 1.

G: Compaq Alpha 21164a 500 MHz, DEC CC. Source: [4], Table 1.

H: “Best-known implementation,” SUNTM UltraSPARCTM. Source: [29], Table 2.

I: DEC Alpha ASM. Source: [37], Table 7.

J: DEC Alpha AXP 21264, DEC CC. Source: [29], Table 1.

K: Hewlett-Packard PA7000. Source: [29], Table 1.

L: Pentium Pro, Java. Source: [35].

M: Texas Instruments TMS320c541 DSP, C. Source: [43].

**Table 3:** Key setup times

	A	B	C	D
CAST	11412	4300	4333	60002
CRYP	758	955	744	21416
DEAL	108396	4000	8635	79709
DFC	23914	7200	7166	90011
E2	7980	2100	9473	136499
FROG	3857000	1386000	1416182	
HPC	234326	120000	120749	1384217
LOKI	22756	7500	7430	
MAG	1490	50	30	208
MARS	4708	4400	4316	54427
RC6	5186	1700	1632	40011
RIJN	17742	850	305	26642
SAFR	4708	4000	4278	38598
SERP	13154	2500	2402	28913
TWOF	18846	8600	8414	88751

Column headings:

A: Linux/GCC-2.7.2.2/Pentium 133 MZh MMX. Source: [5], Table 3.

B: Intel Pentium Pro, C. Source: [37], Table 2.

C: Intel Pentium Pro/II, C. Source: [20], Table 1.

D: Texas Instruments TMS320c541 DSP, C. Source: [43].

**Table 4:** Smart card space requirements

	RAM1	RAM2	ROM
CAST		60	6000
CRYP		52	2000
DEAL		50	2000
DFC		100	2000
E2	300		2000
FROG	2300		
HPC	2000		
LOKI	400		8000
MAG			
MARS	195	60?	3000
RC6	210		1000?
RIJN		52	1000
SAFR		50	2000
SERP		50	1000
TWOF		60	1400

**Table 5:** Security margins

	R	M1	M2	S1	S2
CAST	48	40	20	20 %	140 %
CRYP	12	11	10	9 %	20 %
DEAL	6	10	6	–40 %	0 %
DFC	8	9	6	–11 %	33 %
E2	12	10	9	20 %	33 %
FROG	8				
HPC	8				
LOKI	16	38	16	–56 %	0 %
MAG	6	11	6	–44 %	0 %
MARS	32	20	12	60 %	166 %
RC6	20	21	16	–5 %	25 %
RIJN	10	8	6	25 %	66 %
SAFR	8	7	5	14 %	60 %
SERP	32	17	15	88 %	113 %
TWOF	16	14	6	14 %	166 %

**Table 6:** Operations used

	TabLoo	FixShi	VarShi	Logica	AddSub	MudiSq
CAST	Y		Y	Y	Y	
CRYP	Y	Y		Y		
DEAL	Y	Y		Y		
DFC	Y			Y	Y	Y
E2	Y	Y		Y		Y
FROG	Y				Y	
HPC		Y		Y	Y	Y
LOKI	Y	Y		Y	Y	
MAG	Y					
MARS	Y		Y	Y	Y	Y
RC6			Y	Y	Y	Y
RIJN	Y	Y				
SAFR	Y	Y			Y	
SERP		Y		Y		
TWOF	Y	Y			Y	

Column headings:

TabLoo = Table lookup.

FixShi = Fixed shift or rotate.

VarShi = Data-dependent shift or rotate.

Logica = Logical.

AddSub = Addition or subtraction.

MuDiSq = Multiplication, division or squaring.

**Table 7:** Parallelism and optimal theoretical performance

	Crit1	Crit2	Par
CAST	316		
CRYP	102	85	7
DEAL	1632		
DFC	232		
E2	120	210	4
FROG	1796		
HPC	386		
LOKI	500		
MAG	745		
MARS	258	214	2
RC6	185	181	2
RIJN	86	71	7
SAFR	265		
SERP	556	526	2
TWOF	166	162	3

**Table 8:** 8 bit speeds

CAST	26000
CRYP	12000
DEAL	233000
DFC	35000
E2	6300
FROG	17900
HPC	35000
LOKI	8000
MAG	55000
MARS	5000
RC6	13900
RIJN	3100
SAFR	80000
SERP	34000
TWOF	26500

**Table 9:** Memory usage

	RAM	ROM
CAST	2260	29000
CRYP	800	13979
DEAL	4355	20043
DFC	632	11147
E2	880	275857
FROG	576	14100
HPC	15000	44889
LOKI	10240	15956
MAG	464	6088
MARS	456	19719
RC6	480	7800
RIJN	20000	18405
SAFR	320	132000
SERP	1248	38900
TWOF	8000	19181

## Footnotes

1 Certain commercial equipment, instruments, or materials are identified in this paper to foster understanding. Such identification does not imply recommendation or endorsement by the National Institute of Standards and Technology, nor does it imply that the materials or the equipment identified are necessarily the best available for the purpose.

2 2000 clock cycles was arbitrarily chosen as a cutoff point.

## 3. Appendix A. Tables

In the following tables, some candidates will be abbreviated, as follows:

CAST = CAST-256
CRYP = CRYPTON
LOKI = LOKI97
MAG = MAGENTA
RIJN = Rijndael
SAFR = SAFER+
SERP = Serpent
TWOF = Twofish

NIST does not vouch for the accuracy of data not obtained by NIST.

Table 1 provides the encryption speeds of the candidates, measured in clock cycles, for encrypting one 128 bit block with a 128 bit key.

Table 2 provides the ranking of the encryption speeds of the candidates for encrypting one 128 bit block with a 128 bit key. Note that a ranking of 1 denotes the fastest encryption speed.

Table 3 provides the key setup time, in clock cycles, to encrypt a 128 bit block with a 128 bit key.

Table 4 provides information taken mainly from [37]. RAM1 is the RAM requirement in bytes for candidates not supporting on-the-fly subkey generation. RAM2 is the RAM requirement in bytes for candidates supporting on-the-fly subkey generation. Two entries are given for MARS, one for the original version and one for the proposed modification. The ROM column indicates the number of bytes needed for encryption only.

Table 5 provides the actual number of rounds, R, of a candidate for the 128 bit key size. The table also gives the minimum number of rounds needed for security as stated in Ref. [5]; this is denoted by M1. M2 gives the “raw” number of rounds of the best actual or estimated attack (with no additional rounds). S1 and S2 give the security margins associated with M1 and M2, respectively.

Table 6 indicates the operations used by the candidates (Y = used). Source: [12].

Potential for instruction-level parallelism and optimal theoretical performance can be measured, in theory, by the number of clock cycles in a critical path, as defined (e.g., in Ref. [11] or [21]). Table 7 provides two estimates for the number of cycles in a critical path for a 128 bit encryption. The first estimate, Crit1, is from [21]; the second estimate, Crit2, is from [11]. Par is an estimate of the maximum number of processing elements that can be effectively employed in parallel, as given in Ref. [11].

Table 8 provides 8 bit speeds for the candidates, in clock cycles per 128 bit block encryption with a 128 bit key. Source: [3], appendix.

Table 9 provides some memory usage measurements, in bytes, of the candidates in Java. Source: [19].
